# Impact of proteostasis workload on sensitivity to proteasome inhibitors in multiple myeloma

**DOI:** 10.1007/s10238-025-01713-z

**Published:** 2025-05-26

**Authors:** Jindrich Sedlacek

**Affiliations:** 1 Department of Genetics and Microbiology, Charles University and Research Center BIOCEV, Průmyslová 595, 252 50 Vestec, Czech Republic; 2 Institute of Organic Chemistry and Biochemistry of the Czech Academy of Sciences, Flemingovo náměstí 2, 16610 Prague, Czech Republic

**Keywords:** Proteasome bounce-back response, Autophagy, Redox homeostasis, UPR, Heat shock response, VCP/p97

## Abstract

Genomic alterations and enormous monoclonal immunoglobulin production cause multiple myeloma to heavily depend on proteostasis mechanisms, including protein folding and degradation. These findings support the use of proteasome inhibitors for treating multiple myeloma and mantle cell lymphoma. Myeloma treatment has evolved, especially with the availability of new drugs, such as proteasome inhibitors, into therapeutic strategies for both frontline and relapsed/refractory disease settings. However, proteasome inhibitors are generally not effective enough to cure most patients. Natural resistance and eventual acquired resistance led to relapsed/refractory disease and poor prognosis. Advances in the understanding of cellular proteostasis and the development of innovative drugs that also target other proteostasis network components offer opportunities to exploit the intrinsic vulnerability of myeloma cells. This review outlines recent findings on the molecular mechanisms regulating cellular proteostasis pathways, as well as resistance, sensitivity, and escape strategies developed against proteasome inhibitors and provides a rationale and examples for novel combinations of proteasome inhibitors with FDA-approved drugs and investigational drugs targeting the NRF1 (NFE2L1)-mediated proteasome bounce-back response, redox homeostasis, heat shock response, unfolding protein response, autophagy, and VCP/p97 to increase proteotoxic stress, which can improve the efficacy of antimyeloma therapy based on proteasome inhibitors.

## Introduction

Multiple myeloma (MM) is the second most prevalent and poorly treatable hematologic cancer. MM cells are derived from antibody-producing plasma cells and have high substrate loads on their protein degradation machinery because they constantly produce enormous amounts of aberrant immunoglobulins (Ig) and/or free light chains [1–4]. The 26S proteasome is a central point of the ubiquitin–proteasome system (UPS) and the primary site of degradation for approximately 80% of all cellular proteins. This proteolytic complex is crucial for the targeted elimination of redundant, damaged or regulatory proteins. A high rate of protein synthesis imposes a large burden on proteasome-dependent degradation, and approximately 30% of newly produced proteins are promptly degraded in the proteasome [3, 5, 6]. After synthesis, secreted proteins undergo folding and posttranslational modifications in the endoplasmic reticulum (ER) [7]. Therefore, molecular chaperones help client proteins during folding, exerting stringent quality control that permits only proteins that achieve a native conformation to enter the secretory pathway. Nascent proteins that remain misfolded are degraded through a mechanism called ER-associated degradation (ERAD), in which misfolded proteins are ubiquitinated, extracted from the ER to the cytoplasm and degraded in proteasomes [4, 8, 9].

Thus, protein homeostasis, also known as the proteostasis network, is responsible for the elimination of misfolded, redundant or damaged proteins predominantly via the UPS. The accumulation of these protein species is sensed by cells as toxic. Parallel mechanisms, such as autophagy and storage during aggresome formation, cooperate with the UPS to maintain health proteostasis [10, 11]. Highly conserved adaptive responses, such as the heat shock response and the unfolded protein response (UPR), also exist with the aim of restoring proteostasis. The UPR is a protein homeostasis mechanism activated through the accumulation of misfolded proteins within the ER that results in ER stress [8–11]. The final objective of the UPR is to reduce proteotoxicity by decreasing protein synthesis while selectively upregulating chaperones to facilitate protein folding [10, 11]. Typically, Ig chains are secreted in enormous amounts, contribute to pathogenesis, and cause proteotoxic stress. Thus, drugs that disturb proteostasis are highly effective against MM. Therefore, manipulating proteasome expression, assembly or workload alters the proapoptotic response to proteasome inhibitors (PIs), revealing a causal relationship between proteasome stress/ER stress and apoptotic responses [3]. Immunoglobulin overproduction in MM cells leads to an increased demand for proteasomes, making these cells highly dependent on proteasomal activity for maintaining cellular function. This dependency makes cells highly sensitive to drugs that target protein degradation, such as PIs. Consequently, these agents (i.e., bortezomib, carfilzomib, and the orally available ixazomib) are used globally to treat myeloma and significantly prolong patients' lifespans [1, 12]. Therefore, vulnerability to proteasome inhibitors is affected by both parts of proteostasis, i.e., protein synthesis and degradation. [13]. However, several limitations of bortezomib (BTZ) treatment have become evident. In addition to the emergence of peripheral neuropathy, baseline resistance and the development of acquired resistance in the majority of primary well-responding MM patients are significant concerns [12–19]. To overcome BTZ limitations, second-generation proteasome inhibitors (carfilzomib and ixazomib) have been established to improve efficacy and binding specificity while reducing off-target effects [12, 20–23].

An increase in the load to the proteasome during terminal plasma cell differentiation into antibody-producing B cells has been reported. This also correlates with increased sensitivity to PIs compared with nondifferenced preplasma cells [3, 13, 24]. Indeed, PIs significantly reduce antibody production in the context of in vivo data, suggesting greater toxicity of PIs to activated plasma cells, which is associated with lower levels of Ig in plasma [24–26]. Nonlymphoid cancer cells also become more sensitive to PIs when orphan Ig chains are expressed [24]. In MM cells, the levels of both Ig synthesis and degradation significantly correlate with the apoptotic response to PIs and manipulating Ig production alters cell sensitivity [4, 27, 28]. By exploiting human MM cell lines characterized by differential PIs sensitivity, highly sensitive MM cells express lower levels of proteasome subunit genes and have a greater proteasome workload than relatively PIs-resistant cells do. Additionally, the accumulation of polyubiquitinated substrates at the expense of a pool of free ubiquitin, a condition known as proteasome stress, initiates the cellular apoptotic response and increases the sensitivity of cells to PIs-induced toxicity [24, 29–31].

**Fig. 1:**
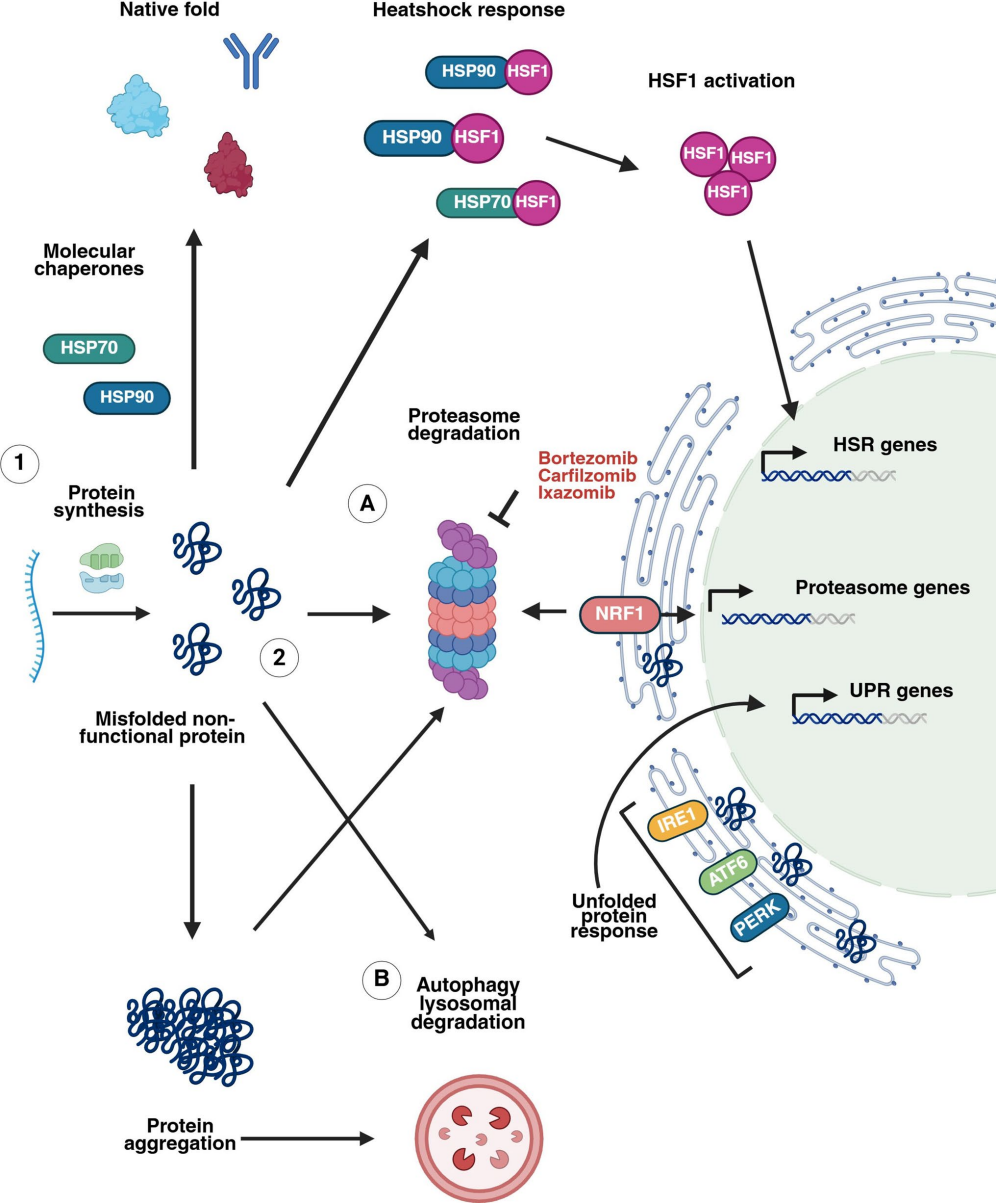
Overview of cellular proteostasis and pathways that restore proteostasis under proteasome impairment. 1. Proteins are synthesized on ribosomes, and molecular chaperones bind misfolded or partially folded proteins and help achieve proper conformation. 2. When proteins are misfolded and/or reach the end of their life or proteostasis is disrupted, two main mechanisms are responsible for protein degradation: *A*, the ubiquitin‒proteasome pathway; and *B*, the autophagy‒lysosomal pathway. A variety of stresses promote protein misfolding and the formation of protein aggregates. The heat shock response is regulated by the transcription factor HSF1. Under physiological conditions, HSF1 is held in an inactive, monomeric state predominantly by HSP90. When misfolded protein species become more abundant, HSF1 can no longer efficiently compete for binding to HSP90 and then undergo trimerization and translocation to the nucleus. When proteasome function is impaired, NRF1 is extracted from the ER membrane, proteolytically processed, and translocated to the nucleus, where it activates proteasome gene expression. When misfolded proteins accumulate in the ER, the unfolded protein response is triggered. Created in BioRender (2025)

The BTZ-adapted cell lines presented increased baseline proteasome activity with a significantly lower rate of protein synthesis. As a result, proteasome inhibition fails to induce proapoptotic ER stress. The combination of reduced protein biosynthesis and enhanced proteasome activity is likely to facilitate cellular homeostasis in the presence of BTZ through a decrease in the production of defective ribosomal products and residual proteasome action [29]. Several studies have described MM cell dedifferentiation and clonal propagation to preplasma progenitors with reduced production of Ig during therapy. Interestingly, extensive Ig production strongly sensitizes myeloma cells to PIs [4, 31–34]. In such cases of BTZ resistance, it is unclear where to focus therapeutic efforts. Therapeutic strategies based on the UPS are generally effective because they still depend on triggering the UPR and ER stress-mediated cell death. This process is difficult to induce in undifferentiated/dedifferentiated or low secretory preplasma/MM cells. Moreover*,* patients receive PIs as a bolus treatment, resulting in quick recovery of proteasome activity, and some cells survive. In contrast, most in vitro studies use continuous treatment so that proteasome activity does not recover [30]. Therefore, several mechanisms and novel therapeutics have been proposed for the exceptional sensitivity of myelomas to proteasome inhibition [8, 16, 22, 34–36]. Currently, understanding the role of proteostasis in promoting effective protein synthesis, folding, secretion, and degradation or identifying new targets within this network has become the subject of extensive research [30, 37–40]. Therefore, this review outlines the current understanding of protein homeostasis and its regulation in MM and provides a rationale for translating advanced knowledge into more effective drug combination therapies (Fig. 1).

## NRF1 and proteasome inhibitors bounce-back response

Transcriptional regulation can determine proteasome abundance and influence proteasome activity. Biogenesis of the 26S proteasome is precisely controlled at multiple steps, such as transcription, particle assembly, and posttranslational modifications [41–43]. The abundance of all 33 distinct 26S proteasome subunits in the cell is regulated by different transcription factors (e.g., NRF1, NRF2, or FOXO 1/4) [44]. Among these factors, the two cap ‘n’ collar basic leucine zipper (CNC-bZIP) transcription factors NRF1 and NRF2 (also known as NFE2L1 and NFE2L2, respectively) are the most significant in mammals. These factors are inducers of proteasome synthesis and are both substrates of proteasome-mediated degradation [14, 42–48]. The ChIP sequencing data demonstrated that the NRF1 and NRF2 target genes overlap slightly. NRF1, not NRF2, predominantly upregulates the expression of all proteasome-related genes in a concerted manner [14, 42, 44]. NRF1 knockdown or knockout suppresses the expression of proteasome genes as well as de novo proteasome formation in response to proteasome impairment. In contrast, NRF2 is a short-lived transcription factor and master regulator of the antioxidant response and the synthesis of lysosomal and anti-inflammatory proteins and some 26S particle components [14, 42, 49]. NRF2 does not induce the expression of proteasome subunit genes in a context-specific manner. These findings suggest a pivotal role for NRF1 in the basal regulation of proteasome genes [42, 44, 45]. Reduced proteasome component expression leads to reduced proteasome activity and results in the accumulation of ubiquitinated proteins. This can be seen in the tissue-specific depletion of NRF1, demonstrating its significant involvement in the regulation of proteostasis. NRF1 levels are also decreased in the most prevalent proteinopathies, for example, in the dopaminergic neurons of Parkinson's disease patients [50–53]. Decreased levels of NRF1 or its proteolytic activator DDI2 result in diminished proteasome activity in a mouse model of Kennedy’s disease, a polyglutamine expansion disorder [54].

Under nonstress conditions, NRF1 is anchored via its N-terminal transmembrane domain into the ER membrane, and the C terminus is extruded into the ER lumen, where it is N-glycosylated. The NRF1 glycoprotein is continuously polyubiquitinated by the ERAD-associated E3 ubiquitin-protein ligase HRD1 and extracted from the ER to the cytoplasm. The extraction of NRF1 from the ER requires the ATPase valosin-containing protein VCP/p97. In cells with functional proteasomes, cytosolic NRF1 is rapidly degraded. However, in cells with insufficient proteasome activity or with partially inhibited proteasome activity, the NRF1 half-life may increase until it is cleaved in the cytosol by the aspartyl protease DDI2, deglycosylated by the peptide N-glycanase 1 (NGLY1), and translocated to the nucleus. Overall, these steps are crucial mechanisms for the regulation of NRF1 activity [42, 44, 55–58]. Nevertheless, coinhibition of chymotrypsin-like and caspase-like proteasome activities suppresses NRF1 activation and sensitizes cancer cell lines to BTZ. This finding likely explains the significantly reduced NRF1-mediated proteasome bounce-back response at higher concentrations of BTZ, which affected all 20S proteolytic activities [58–60]. Only proteolytically processed and deglycosylated NRF1 induces the expression of all proteasome subunits and other target genes by binding with small musculoaponeurotic fibrosarcomas (MAFs) to antioxidant response elements (AREs) within promoters of target genes. Increased proteasome subunits expression leads to increased proteasome biogenesis and to the restoration and enhancement of proteasome functions [42, 44, 57, 61].

Patients receive a BTZ injection, which results in maximum inhibition within one hour followed by rapid recovery of proteasome activity. Replacing continuous treatment with a one-hour pulse significantly increased the differences in sensitivity among a panel of MM cell lines more than tenfold and revealed that more sensitive cell lines undergo apoptosis faster. Clinically achievable inhibition of proteasome active sites was sufficient to induce cytotoxicity in only one cell line [30]. In addition, overexpression of the β5 subunit and rapid recovery of proteasome activity were observed in BTZ-resistant cell lines [29, 62, 63]. These insights also provide a rationale for targeting any key factors in the NRF1 pathway as a master regulator of PIs bounce-back response in MM cells [64–67]. MM cells also exhibit baseline NRF1 activation and strongly depend on DDI2 for survival, and DDI2 knockout is cytotoxic to MM cells both in vitro and in vivo [Recent studies have shown that DDI2 knockout prevents proteolytic processing and nuclear translocation of NRF1, causing impaired proteasome activity recovery upon irreversible proteasome inhibition and thereby increasing sensitivity to PIs. The addition of wild-type DDI2 but not catalytically dead DDI2 fully rescued these phenotypes. [65–70]. The activation of NRF1 via DDI2 contributes to the mechanisms driving BTZ sensitivity, initiating a proteasome bounce-back response, and alterations in the proteolytic functions of DDI2 potentiate the cytotoxicity of PIs. It was also shown that inhibition of NRF1 proteolytic processing by DDI2 via the clinically approved inhibitor of the HIV protease nelfinavir increased PIs susceptibility in treated MM cells [65–67]. Taken together, these findings indicate that blocking the DDI2-NRF1 axis is essential for proteasome activity recovery under impairment and enhances proteotoxicity [66–70]. Additionally, genetic or chemical disruption of NGLY1 deglycosylation activity results in the accumulation of unprocessed and nonfunctional NRF1. Thus, under these conditions, proper NRF1 activation is impaired. Thus, NGLY1 inhibitors strongly potentiate the cytotoxic activity of carfilzomib against T cell-derived acute lymphoblastic leukemia and MM cells [71]. Overall, the important cause of resistance to proteasome inhibition is the bounce-back response, a recovery pathway driven by NRF1, which contributes to maintaining proteasome activity via proteasome subunit resynthesis upon impairment of its function. As a result, inhibiting this pathway potentiates the cytotoxic effect of PIs and could improve treatment outcomes.

## Reactive oxygen species and the antioxidant response

Immunoglobulins produced by MM cells are multisubunit proteins that are kept together by intra- and interchain disulfide bonds as well as different noncovalent interactions, and their folding is oxidative by nature [72]. Molecular oxygen acts as the electron acceptor for each disulfide bond reaction, leading to the production of large amounts of reactive oxygen species (ROS) in Ig-producing cells. In mature plasma or MM cells, proper folding of one Ig molecule requires the formation of approximately 10^2^ disulfide bonds. Thus, one plasma cell is capable of synthesizing thousands of Ig molecules per second, indicating that one Ig-producing cell may produce approximately 10^5^ disulfide bonds at the same time [73–75]. To neutralize the increased ROS levels caused by Ig production, plasma cell maturation is associated with an increased antioxidant response [76, 77]. Cells usually protect proteins via an increased antioxidant response or repair oxidized proteins to restore their functions. If these mechanisms fail, oxidized proteins may become cytotoxic. Therefore, MM cells are dependent on the suppression of oxidation or the removal of oxidatively damaged proteins. Numerous studies have demonstrated that the proteasome plays a pivotal role in the recognition and degradation of oxidized proteins [73–76, 78]. In MM cells, where a high rate of Ig synthesis is a significant factor contributing to the high production of ROS, further induction of oxidative stress or inhibition of the antioxidant response can be an additional therapeutic strategy. Inhibition of the proteasome leads to the accumulation of unfolded proteins, which trigger endoplasmic reticulum (ER) stress and further accelerate oxidative stress [28, 74–79]. Unresolved ER stress, which is considered an important mediator of PIs cytotoxicity, has been shown to cause cell death via multiple pathways, including overproduction of ROS [28, 75, 76]. BTZ-induced cytotoxicity can be dampened when cells are supplemented with cysteine or its derivative, glutathione (GSH), a major component of the cell's antioxidative defence. Increasing intracellular GSH levels fully abolished bortezomib-induced cytotoxicity, and NRF2-mediated transcriptional changes [80]. In a metabolomic study of AMO-1-derived resistant cells to proteasome inhibitors, the glutathione synthesis pathway was highly enriched [81].

In fact, oxidative stress has been identified as one of the mechanisms of BTZ-induced cytotoxicity in MM and nonmyeloma cell lines [82, 83]. ROS generation also precedes the initiation of the BTZ-induced apoptotic cascade [84, 85]. Furthermore, cotreatment with antioxidants rescues PIs-induced ROS generation and suppresses cell death [80, 86]. Cotreatment of MM cells with carfilzomib and panobinostat, a broad spectrum of histone deacetylase inhibitors, resulted in ROS-dependent cell death [87]. Panobinostat has been approved for the treatment of patients with relapsed and refractory MM [88]. Although several mechanisms have been suggested for panobinostat, the primary mechanism of action is likely dependent on the generation of ROS [89, 90].

The general stress response transcription factor NRF2 is a major regulator of the antioxidative response and detoxification, is implicated in chemoresistance, and contributes to malignant phenotypes through its effects on proliferation. NRF2 functions to rapidly alter the sensitivity of cells to oxidants and electrophiles via the transcriptional activation of a broad spectrum of antioxidant and drug metabolism genes [91, 92]. Under physiological conditions, NRF2 is inactive in the cytoplasm in complex with KEAP1, which facilitates the ubiquitination of NRF2 and ensures its subsequent proteasomal degradation. Modification of KEAP1 cysteine residues by oxidants reduces its affinity for NRF2, resulting in a decrease in NRF2 ubiquitination and stabilization [91, 92]. Accumulated NRF2 is translocated into the nucleus, where it forms complexes with small MAFs, binds together to responsive elements, and enhances the transcription of target genes [91]. The expression of genes related to bortezomib resistance revealed enrichment of NRF2 target genes, which are activated as the main component of the antioxidant response pathway [83, 92]. NRF2 controls basal expression and ensures the induction of genes encoding antioxidant and detoxifying enzymes, including heme oxygenase-1, NAD(P)H:quinone oxidoreductase-1, superoxide dismutase, catalase, glutathione *S*-transferase, and glutamate cysteine ligase [94, 95]. Increased levels of antioxidant-related pathway genes have been linked with drug resistance in different tumors, including resistance to BTZ in samples from patients with B cell derivative mantle cell lymphoma [96]. Increased intracellular GSH levels block BTZ-induced NRF2-associated stress responses, including the upregulation of the light-chain subunit xCT of the Xc- cystine-glutamate antiporter, which is necessary for GSH synthesis [80].

NRF2 activation also results in increased translation of the autophagy receptor sequestosome 1 (SQSTM1)/p62, an autophagosome cargo protein that targets ubiquitin-labeled proteins for selective autophagy [94, 97]. NRF2 also increases the cellular capacity to degrade oxidized proteins, which is due to increased expression of the 20S proteasome and the Pa28αβ (11S) proteasome regulator [98]. NRF2 also regulates the expression of the proteasome maturation protein (POMP), a chaperone responsible for the assembly of active proteasome particles from different inactive subunits. POMP was also described as a mediator of the BTZ-resistant phenotype in MM [99]. Overall, NRF2 is constitutively activated in approximately 50% of MM primary samples and in MM derivative cell lines. Moreover, genetic inhibition of constitutively expressed NRF2 reduces MM cell viability. These findings confirmed that PIs induced the expression of NRF2 in MM cell lines and primary cells. Furthermore, genetic inhibition of NRF2 in PIs-treated cells increased ER stress through the regulation of CCAAT-enhancer-binding protein homologous protein (CHOP), and the combination of the NRF2 potent inhibitor all-trans retinoic acid (ATRA) with BTZ highly resensitizes resistant cells to BTZ in preclinical models [100]. Therefore, NRF2 was proposed to be a promising target for combination therapy with PIs. Recent studies have identified new functions of NRF2 in the regulation of metabolism, mitigation of ER stress, and other essential cellular processes, establishing NRF2 as a pleiotropic transcription factor. The potential of targeting NRF2 in multiple myeloma to overcome bone marrow microenvironment-mediated drug resistance has also been discussed in detail [101]. Not surprisingly, the reemergence interest in NRF2 has prompted many studies to elucidate its role in various types of malignancies and investigate possible therapeutic strategies to prevent or counteract its activation [102–104].

In addition, several redox-targeted drugs are in development or are currently used in the clinic for the treatment of different cancers [105, 106]. Decrease in glutathione synthesis induced by L-buthionine-sulfoximine synergizes with BTZ. On the other hand, antioxidants such as N-acetyl-L-cysteine and ascorbic acid protect MM cells from BTZ-mediated toxicity [80, 107]. Nitric oxide (^•^NO), a highly reactive free radical-generating prodrug, JS-K, upon reaction with glutathione, synergizes with the antimyeloma activity of BTZ and demonstrates promising preclinical activity in MM models [108]. These findings demonstrate a strong connection between redox homeostasis and sensitivity to proteasome inhibitors. The repurposing of drugs that target glutathione metabolism, such as ezatiostat and canfosfamide, may benefit the treatment of myeloma patients.

## Heat shock response

A large class of molecular chaperones known as heat shock proteins (HSPs) **c**onstitute the first line of cellular protection against various stressful conditions. These proteins play key roles in de novo folding, assembly of complexes, and protein sorting. The stress-driven synthesis of HSPs is a universal phenomenon that occurs in all eukaryotes and prokaryotic cells and plays a crucial role in preserving protein homeostasis [109]. Blocking this step is highly toxic, especially to myeloma cells, by causing the UPR and simultaneously increasing the substrate load on proteasomes, resulting in high proteotoxic stress. Compared with the majority of somatic cells, MM cells are highly dependent on the HSPs chaperone machinery because of the excessive load of misfolded proteins, and exposure to PIs strongly induces the expression of HSPs [33, 110–113]. Proteasome inhibition causing the accumulation of misfolded proteins, and this stress is primarily compensated by chaperones, predominantly those in the HSP70 family [114, 115].

Therefore, HSPs help reduce proteotoxic stress, prevent the terminal phase of the UPR, and support MM survival. HSPs also play important roles in cellular differentiation, proliferation, and carcinogenesis. Cancer-transformed cells differ from normal cells in that they have a greater need for these chaperones for survival [111–113]. The transcription factor—heat shock factor 1 (HSF1) controls the expression of HSPs and has been investigated as a potential therapeutic target in MM [110, 113]. It has also been identified as a promising anticancer target because of its critical role in cancer cell growth, metastasis, and survival [116]. In preclinical studies, shRNA-mediated HSF1 silencing and HSF1 inhibitors (e.g., CCT251236 and KRIBB11) were shown to induce cytotoxicity through the induction of the UPR and to lead to BTZ sensitization [110, 117]. A member of the heat shock protein 70 family (HSC70) is essential for chaperone-mediated autophagy and drives the rerouting of misfolded proteins for subsequent degradation [118–120]. In contrast, HSP90 is responsible for stabilizing inherently catastrophic gene mutations in the oncogenome to prevent protein degradation and cellular apoptosis, promoting the early malignant transformation and propagation of cancer cells. This buffering of substantial mutations may result in the development of drug resistance, resulting in additional accumulation of mutations in the genome. As a result, when HSP90 is overwhelmed, the manifestation of mutant phenotypes is likely to result in the selection of phenotypes that are beneficial for cancer survival [121]. Therefore, HSP90s and HSP70s are prospective targets and the most investigated HSPs in cancer biology, as they play a role in the folding of proto-oncogenes [122]. Specifically, HSP90s stabilize mutant p53, thereby allowing cancer cells to evade growth-stopping signals [121, 123]. In preclinical models, the induction of HSP70 and HSP90 results in strong UPR activation and subsequent cell apoptosis [114, 124, 125].

The combination of the HSP90 inhibitors tanespimycin, KW-2478, and retaspimycin with BTZ had synergistic effects on toxicity in vitro [126–128]. Several other HSP90 inhibitors, such as NVP-HSP990, SNX5422, NVP-AUY922, and PU-H71, have shown promising preclinical results against MM [129–132]. Many of these HSP90 inhibitors have completed phase I clinical trials. Unfortunately, their narrow therapeutic index and modest clinical significance have limited further clinical use [133, 134]. When HSP90 inhibitors were tested as monotherapies in phase I clinical trials, only retaspimycin displayed modest anti-MM activity, suggesting that a deeper understanding is crucial for overcoming drug resistance in the clinic [135]. Tanespimycin, a derivative of the antibiotic geldanamycin, was proven to be effective against MM in vitro and produced encouraging outcomes in phase I/II clinical trials **(**Table 1**)**, in combination with BTZ [136–138]. The HSP70 inhibitors JG98, JG342, MAL3-101, PER-16, and VER-155008 have been created as alternative therapeutic agents to HSP90 inhibitors with promising preclinical activity and the ability to overcome PIs resistance [115, 124, 125, 132, 139, 140]. Compared with control siRNA, the silencing of individual HSP family members significantly increased BTZ-induced apoptosis. However, the rate of apoptosis observed after the silencing of individual HSPs combined with BTZ was significantly lower than that observed after the combination of BTZ and HSF1 silencing [110]. These findings demonstrate the strong dependency of MM cells on the heat shock response for survival and support the evaluation of different pharmacological targets in this pathway in myeloma.

**Table 1:** Drugs targeting proteostasis in MM

Drug	Mechanism of action	Current status	Study design	Clinical trial identifier/PMID
HIV protease inhibitor				
Nelfinavir	Inhibition of proteasome backbone response via NRF1, block of AKT phosphorylation, triggers proapoptotic PERK signaling	Phase 1, completed	Nelfinavir + BTZ + metformin in patients with relapsed and/or refractory MM	NCT03829020
		Phase 1, completed	Nelfinavir + BTZ in patients with relapsed or progressive advanced hematological cancer	NCT01164709
		Phase 2, completed	Nelfinavir + BTZ + dexamethasone, as BTZ-sensitizing drug in patients with proteasome inhibitor-nonresponsive myeloma	NCT02188537
		Phase 1/2, terminated	Nelfinavir + lenalidomide + dexamethasone in progressive MM	NCT01555281
Pro-oxidant drugs				
JS-K	NO generation, cytotoxicity in sensitive and resistant MM cell lines, as well as patient-derived MM cells. Apoptosis associated with PARP, caspase-8/9 activation, Bcl-2 phosphorylation, cytochrome c, apoptosis-inducing factor, and endonuclease G release	Preclinical	N/A	17,384,201 [108]
Canfosfamide	GSH analog that sensitizes cancer cells to the cytotoxic effects of chemotherapies, increase cellular ROS, apoptosis	Phase 2, completed	Intravenous infusion of canfosfamide every 2 weeks for MM, mantle cell lymphoma, diffuse large B cell lymphoma	NCT01148108
Heat shock response				
CCT251236 and KRIBB11	Inhibition of HSF1, induce caspase-mediated cell death that was associated with an increase in EIF2α phosphorylation, CHOP expression, BTZ sensitization	Preclinical	N/A	27,487,129 [110]
Tanespimycin	HSP90 inhibitor, UPR, apoptosis	Phase 3, completed	Tanespimycin + BTZ in patients with MM in first relapse	NCT00546780
KW-2478	HSP90 inhibitor, enhancing apoptosis, decrease in IgH locus translocation products as FGFR3, reduction of c-Maf and cyclin D1 expression	Phase 1, completed	KW-2478 in patients with relapsed/refractory MM for determination of toxicity and dosing	NCT00457782
		Phase 1/2, completed with results	KW-2478 + BTZ in subjects with relapsed/refractory MM	NCT01063907
IPI-504	HSP90 inhibitor	Phase 1, completed	IPI-504 in patients with relapsed and relapsed refractory MM	NCT00113204
SNX-5422	HSP90 inhibitor	Phase 1, completed	SNX-5422, in subjects with refractory hematological malignancies	NCT00595686
MAL3-101	HSP70 inhibitor, XBP1 mRNA splicing, apoptosis and cell cycle arrest, BTZ sensitization/synergic effect	Preclinical	N/A	21,977,030 [125]
VER-155008 and JG98	HSP70 inhibitors, sensitization to PIs, disturbation of UPR and autophagy	Preclinical	N/A	36,170,818 [115]
Autophagy				
Everolimus	mTOR inhibitor, activation of mTOR-autophagy axis	Phase 1, terminated	Everolimus + lenvatinib in patient with recurrent plasma cell myeloma, everolimus + Ponatinib in patient with refractory plasma cell myeloma	NCT03878524
		Phase 1, completed	Everolimus + bendamustine in patients with relapsed/refractory hematological malignancies	NCT02240719
		Phase 1/2, completed with results	Panobinostat + everolimus in patients with recurrent MM	NCT00918333
		Phase 1, terminated with results	Everolimus + pomalidomide + dexamethasone in patients with relapsed/refractory MM	NCT01889420
Nab-rapamycin	mTOR inhibitor, activation of mTOR-autophagy axis	Phase 1, withdrawn	Nab-rapamycin + pomalidomide + dexamethasone for relapsed and refractory MM	NCT03657420
Chloroquine	Inhibits autophagy by inhibiting autophagosome and lysosome fusion	Phase 2, terminated with results	Chloroquine in combination with BTZ and cyclophosphamide for relapsed and refractory MM	NCT01438177
Hydroxychloroquine		Phase 1, completed	Carfilzomib + hydroxychloroquine in patients with relapsed/refractory MM	NCT04163107
		Phase 1, completed	hydroxychloroquine + BTZ in patients with relapsed or refractory MM	NCT00568880
3-Methyladenine (3-MA)	Autophagy inhibitor via effect on class III Phosphoinositide 3-kinases	Preclinical	N/A	19,509,276 [147]
UPR axis				
NMS-03597812	An orally bioavailable inhibitor of PERK with potential antineoplastic activity	Phase 1, terminated	Patients receive NMS-03597812 administered orally as single agents or in combination with dexamethasone in patients with relapsed or refractory MM	NCT05027594
VCP/p97				
CB-5083	VCP/p97 inhibition, UPR induction and apoptosis, block of NRF1 signaling	Phase 1, terminated	CB-5083 + dexamethasone, in patients with relapsed and refractory MM	NCT02223598
Disulfiram	Suppress forming of VCP/p97 complex with adaptor NPL4, oxidative stress, block of NRF1 cleavage	Phase 1, terminated	Disulfiram in combination with copper gluconate in patients with treatment-refractory MM	NCT04521335

The table shows promising drugs in advanced preclinical or early clinical phases of development

## Autophagy

Autophagy “self-eating” is a mechanism through which proteins and other damaged, dysfunctional, or unneeded parts of the cell are eliminated in the lysosome. Autophagy is typically considered a prosurvival mechanism, and autophagic cell death, which occurs via the activation of autophagy, has also been described. These findings demonstrate the complexity of this degradation pathway. Studies have shown that autophagy, the UPS, ERAD, the UPR, and the heat shock response are closely related. Uncovering the roles of the genes essential for autophagy has led to a new era in our understanding of the origination and progression of human diseases and has shed light on novel therapeutic targets in this pathway [141, 142]. Autophagy also maintains the quality control of newly synthesized proteins, potentially explaining the high levels of basal autophagy in MM, characterized by high levels of protein synthesis [27, 39, 142]. Therefore, this process is essential for MM survival as an alternative pathway for protein degradation. However, many agents with anti-MM activity, such as the TOR kinase complex 1 (mTOR1) inhibitor rapamycin, which is used as an immunosuppressant and BTZ itself, have been shown to induce autophagy. The mTOR1 inhibitor everolimus was used as a single agent in a phase 1 trial in patients with relapsed or refractory MM. Among the fifteen patients assessed, ten experienced a positive clinical outcome, with one patient achieving partial remission after four therapeutic cycles [143]. A phase 1 trial also assessed the safety and efficacy of everolimus in combination with lenalidomide in patients [144]. These promising outcomes highlight the potential use of mTOR1 inhibitors for the treatment of relapsed or refractory MM. Additionally, several clinical trials focusing on the role of mTOR1 inhibitors, such as everolimus or nab-rapamycin, have been conducted (Table 1). However, mTOR1 inhibition and subsequent autophagy activation may represent a possible PIs toxicity escape mechanism [145–147]. Whether mTOR could be a drug target is moderately controversial because mTOR also regulates other critical cellular processes in addition to autophagy [148].

Cell-based studies have revealed that SQSTM1/p62 helps maintain protein homeostasis in MM cells by clearing redundant and misfolded proteins. Nevertheless, SQSTM1/p62 is increased after PIs treatment, suggesting that this adaptor protein plays a role in the escape mechanism from PIs-induced toxicity and that SQSTM1/p62 targeting have the potential to overcome resistance [147]. Consistently, knocking down SQSTM1/p62 increases sensitivity to PIs, suggesting that the induction of this gene may be a novel molecular target for overcoming PIs resistance in MM [149, 150]. The inhibition of SQSTM1/p62 in resistant cells also sensitizes cells to PIs [149]. In addition, recent studies have shown that NRF1 is involved in the transcriptional regulation of compensatory autophagy mechanisms upon proteasome insufficiency. In response to PIs, NRF1-deficient cells exhibit profound defects in invoking autophagy and clearing aggresomes. The same results were also observed in NGLY1 knockout cells, in which NRF1 is nonfunctional [151, 152]. These novel findings provide additional rationale for repurposing nelfinavir. Additional studies have revealed increased expression levels of high mobility group box 1 (HMGB1), an important regulator of autophagy, in BTZ-resistant cells [153]. In the same study, BTZ was administered with the autophagy blocker lycorine to resensitize MM cells, and its effect was enhanced both *in vitro* and *in vivo*. However, HMGB1 alone is unlikely to be responsible for driving BTZ resistance, as the regulation of autophagy is a highly complex process [153]. It has also been reported that cysteine-rich protein 1 (CRIP1) promotes PIs resistance in MM dually via the UPS through stabilization of the proteasome activator PA200 and promotion of autophagy [154]. Other mechanisms of BTZ resistance in the context of autophagy include the upregulation of the cytoskeleton protein profilin-1, which promotes autophagy by binding to the Beclin-1 complex [155]. The dual inhibition of the proteasome and autophagy with BTZ in combination with hydroxychloroquine has recently emerged as a prospective strategy for overcoming resistance to proteasome inhibitors. Hydroxychloroquine also has a synergistic effect when used in combination with carfilzomib [156, 157]. Bafilomycin A1, a reversible inhibitor of vacuolar-type ATPase (V-ATPase) that inhibits the late phase of autophagy, also potentiates the anti-MM activity of BTZ by inducing a high level of ER stress [158]. Crucially, given the complexity of the autophagic pathway as well as the nonselective targeting of autophagy or combined inhibition of autophagy and the proteasome in preclinical studies, highly variable and conflicting results, ranging from synergistic to antagonistic effects, have been reported [144–147, 149, 156, 157, 159]. For example, two specific autophagy blockers, chloroquine and 3-methyladenine, have shown antagonistic effects when they are combined with BTZ [147].

## ER stress and UPR signaling

Endoplasmic reticulum governs protein folding and modification through various chaperones and enzymes that reside in this cellular compartment. The perturbation of protein folding, export, and degradation, for example, caused by proteasome inhibitors, leads to an overwhelmed ER capacity and subsequent accumulation of unfolded proteins in the ER. This cellular state is referred to as ER stress. In turn, ER stress induces evolutionarily conserved signaling cascades, named the unfolded protein response, which reinstate ER homeostasis through the suppression of global protein synthesis, enrichment of protein folding machinery, and enhancement of protein degradation via ERAD [160–163]. The UPR is activated via tripartite transmembrane stress detection systems. Specifically, eukaryotic translation initiation factor 2 alpha kinase 3 is also known as protein kinase R-like endoplasmic reticulum kinase (PERK), inositol-requiring enzyme 1 (IRE1), and activating transcription factor 6 (ATF6) [161–163]. Named UPR switchers are maintained in an inactive state via interactions between their ER lumen domains and the binding immunoglobulin protein (BiP), the ER-resident chaperone from the HSP70 protein family. When unfolded proteins accumulate in the ER and induce ER stress, the interaction between BiP and these client proteins is destabilized, allowing dimerization and autocatalytic activation of these sensors. If ER homeostasis is not restored, chronic UPR activation results in cell death [162–165]. MM and mature plasma cells are characterized by the synthesis and secretion of excessive levels of Ig and its folding into tertiary structures in the ER, where the UPR maintains equilibrium between the rate of protein biogenesis and the ability to fold and degrade nascent proteins. UPR activation under proteotoxic stress also results in the translation of chaperone proteins involved in global protein folding [162–165]. The main function of the UPR consists of the upregulation of genes involved in ER function and in the suppression of the global production of polypeptides that cannot be correctly processed or degraded. Thus, restoring and/or increasing the capacity of the ER to fold and process proteins [166]. Therefore, continual overproduction of Ig makes MM heavily dependent on the UPR axis for survival [162–168].

## Inhibition of PERK

Protein kinase R-like ER kinase (PERK) is a transmembrane protein that resides in the ER and acts as an essential sensor of ER stress. The activated PERK C-terminal cytosolic domain has serine/threonine kinase activity. Upon the accumulation of unfolded proteins in the ER lumen, PERK dimerizes, and subsequent phosphorylation inactivates eukaryotic initiation factor 2 alpha (eIF2α), which is responsible for 80S ribosome complex assembly. This results in the inhibition of total protein synthesis and the suppression of acute proteotoxic stress [169–172]. eIF2α is also required for the selective translation of several mRNAs, such as those that encode activating transcription factor 4 (ATF4), which activates many genes involved in the UPR and integrates the stress response [172, 173]. In addition, the stress response coordinated through PERK can both promote and/or inhibit tumorigenesis and regulate cell death. Therefore, PERK has been thoroughly investigated in many cancer types [174–176]. However, inactivation of PERK sensitizes cancer cells to a broad spectrum of chemotherapy drugs [177–182], whereas knockdown of PERK in MM cells results in an autophagic type of cell death [182]. In the BTZ-resistant subpopulation of myeloma cells, resistance can be reversed by perturbation of eIF2α [183]. Consistent with these findings, silencing of the PERK gene significantly enhanced cell death, emphasizing the importance of PERK in MM biology. Moreover, GSK2606414, an ATP-competitive potent PERK inhibitor, had significant antiproliferative and proapoptotic effects on a broad panel of MM cell lines alone, and in combination with BTZ exerted additive toxic effects, probably through increased proteotoxic stress [171, 180, 182]. The previously mentioned HIV protease inhibitor nelfinavir triggered only a partial ER stress response characterized by the absence of detectable PERK and ATF6 activation and/or the accumulation of misfolded proteins within the ER. However, nelfinavir disturbs proteostasis via the modulation of eIF2α dephosphorylation, affecting the translation rate [184]. Overall, PERK inhibition may represent a new combinatorial therapeutic strategy, not only for MM.

## XBP1 and load to the proteasome

Transcription factor X-box binding protein (XBP1) is a key effector of the mammalian UPR whose activity is activated by the action of inositol-requiring enzyme (IRE1). IRE1 is an ER-resident kinase/ribonuclease that transduces ER stress. This enzyme removes an intron from the XBP1 mRNA (sXbp1), leading to the synthesis of a potent transcription factor that further regulates a broad spectrum of genes involved in preserving ER homeostasis [185–187]. Active XBP1, together with another ER-resident transcription factor, ATF6, induces lipid biogenesis to maintain ER expansion, induces chaperone expression to support nascent ER protein folding, and alternatively initiates the ERAD pathway to decrease ER stress [186]. XBP1 is essential for highly secretory cells [187, 188]. Therefore, XBP1 is also expressed at greater levels in plasma and MM cells than in B cell-derived cell lines and other developmental stages, such as progenitor, pre-B, and mature B cells [189]. XBP1-deficient mice exhibit abnormal plasma cell production and Ig secretion after immunization. However, these cells exhibit a normal cell proliferation rate and germinal center formation, suggesting that XBP1 is a key factor for normal B cell differentiation [189, 190].

XBP1 is expendable for terminal plasma cell differentiation, as indicated by the proportionable level of affinity-matured plasma cells in XBP1-deficient mice versus control mice [191]. However, the level of Ig is significantly reduced in cells lacking XBP1, which highlights its importance in the regulation of antibody secretion [191]. However, the level of Ig is significantly reduced in cells lacking XBP1, which highlights its importance in the regulation of antibody secretion [191]. However, the development of memory B cells, which do not secrete antibodies, is also unaffected by XBP1 [34, 192]. Interestingly, RNA-seq analysis of XBP1-deficient plasma cells in a tamoxifen-inducible system revealed that the expression of 632 genes was disrupted compared with that in plasma cells with a normal XBP1 status, including a global reduction in all transcripts encoding Ig heavy chains [193]. Other studies have shown that Xbp1 is more highly expressed in myelomas than in normal plasma cells [194–197]. XBP1 is also required for the differentiation of B cells into mature plasma cells, long-lived terminally differentiated B cells that are responsible for the production of antigen-specific Ig, and has been shown to be involved in BTZ sensitivity [197, 198]. XBP1 activation via UPR inducers (e.g., tunicamycin and dithiothreitol) promotes differentiation into cells with mature plasma characteristics, as it reduces the nucleocytoplasmic ratio and increases the production of Ig. siRNA-mediated knockdown of XBP1 also disrupts MM cell maturation, and a low level of XBP1 is a characteristic feature of the myeloma progenitor cell population [34, 199].

Low-density XBP1-expressing (or XBP1-depleted) plasma and MM cells are less differentiated and exhibit significantly lower Ig production, reduced ER stress, and a decreased load on the proteasome [196–199]. The overexpression of spliced sXbp1 in primary MM correlates with poor overall survival, and the increased expression level of sXbp1 in B cells mimics the MM phenotype in mice. These findings strongly suggest that sustained IRE1-mediated activation of XBP1 may significantly contribute to MM pathogenesis [34, 197–199].

Increasing evidence of the role of XBP1 in MM pathogenesis has revealed its potential application as a prognostic marker and therapeutic target [176, 194–198]. Additionally, the majority of bone marrow plasma cells presented increased XBP1 levels, indicating sensitivity to BTZ. However, subpopulations of cells with lower Ig production status can be detected, and it is hypothesized that these subpopulations with downregulated XBP1 are responsible for possible relapse after therapy [34, 195]. MM cells dedifferentiation and clonal propagation of preplasma cells has also been reported during therapy [32, 33]. Despite extensive research on MM, its broad disease heterogeneity, omics landscape, and pattern of clonal evolution are still poorly understood, hampering efforts toward improved treatment [200, 201]. Treatment-resistant MM patients can exhibit expansion of low XBP1 status in tumor cells and preplasmablasts, which are intrinsically resistant to BTZ owing to decreased Ig production and decreased proteasome workload [34, 202]. Cell-based studies have shown that low sXbp1 production and low Ig abundance are correlated with poor responses to BTZ therapy and decreased sensitivity [203–205].

XBP1 low-status or XBP1-negative cells, which correspond to B cells or preplasmablasts, are detected in BTZ-resistant cell populations and are significantly enriched in refractory MM isolates that survive BTZ therapy. A subpopulation of sXbp1-negative cells produces lower amounts of Ig and has fewer UPR markers, indicating reduced ER stress and less dependency on the UPR pathway in sXbp1-negative cells than in cells with an unaffected status [34, 202–204, 206]. MM patients with high secretory Ig levels respond better to BTZ than do patients with low Ig secretion [3, 206, 207]. An inactivating mutation in the Xbp1 splicing domain, c.499C > A (L167I), has also been identified in MM patients and may confer resistance to PIs [208, 209]. Differences in sensitivity to PIs can thus be explained by various ratios of the amount of Ig to the number of active proteasomes in the cell (load). Unfortunately, the degree of plasma cell differentiation and secretory status have not yet been considered in clinical studies [3, 4]. These findings reveal the importance of determining the XBP1 status prior to BTZ treatment because its potential effect on BTZ sensitivity may be beneficial for predicting treatment response and patient prognosis [206, 210].

## VCP/p97

The ATPase VCP/p97 and its main adaptor proteins, such as NPL4 and/or UFD1, are crucial components of the UPS; these proteins extract and disassemble substrates from different cellular locations, regulate various steps in autophagy, and are involved in the protein secretory pathway. VCP/p97 is highly evolutionarily conserved, representing approximately 1% of the total cellular protein content, and has a central function in endoplasmic reticulum-associated degradation (ERAD), mitochondria-associated degradation, aggresome formation, endosomal trafficking, and ribosome-associated quality control [211–217]. Because VCP/p97 has a different set of substrates at various cell sites, its substrate specificity is determined by approximately 40 adaptor proteins, which link VCP/p97 to ubiquitinated substrates through specific VCP/p97 interaction domains and ubiquitin-binding domains (UBDs) [218, 219]. For example, misfolded proteins from the ER are retrotranslocated to the cytosol in a VCP/p97-UFD1-NPL4-dependent manner. When the activity of VCP/p97 is insufficient, ERAD substrates accumulate and trigger the UPR [220]. Despite the use of PIs in the clinic, the development of resistance to therapies is very common, and PIs are not effective in solid tumors [221–223]. In contrast, VCP/p97 inhibitors are highly effective in both blood and solid cancers. In addition, not all VCP/p97 substrates are targeted for proteasome degradation, further supporting reports that VCP/p97 acts upstream of both degradation systems and participates in the activation of cell death [220–225]. Therefore, inhibition of VCP/p97 has been proposed as an alternative approach for treating MM patients who relapse after therapy with PIs [226, 227]. Preclinical studies on the orally bioavailable VCP/p97 inhibitor CB-5083 have demonstrated robust activity in highly secretory myeloma cells and several in vivo MM models. However, only minor toxicity was observed in untransformed, nonsecretory control cells. The synergistic anti-MM activity of CB-5083 and PIs can likely be explained by the VCP/p97-mediated retrotranslocation of the transcription factor NRF1, which affects the expression of proteasome subunit genes in the case of proteasome activity insufficiency. However, a phase I clinical trial of CB-5083 was stopped because of off-target effects and high toxicity [42, 222, 226, 228]. The VCP/p97 ATPase inhibitor OSSL_325096 induced apoptosis in MM cell lines, including BTZ-resistant cells, and in patient-derived primary myeloma cells via ER stress and associated signaling [229]. The disulfiram metabolite copper bis(N,N-diethyldithiocarbamate), which is responsible for the anticancer effects of disulfiram, also inhibits the function of VCP/p97 in the cytoplasm. This process is mediated by the release of cupric ions under prooxidative conditions, disrupting the zinc finger motifs of the adaptor protein NPL4 and locking it into the essential conformational switch of the complex [210, 230, 231]. This compound also blocks translocation from the ER and cytoplasmic processing of NRF1 and has cytotoxic effects on a broad panel of PIs adapted and naive myeloma cell lines as well as on cells derived directly from patient samples with MM before and after BTZ therapy [230, 232]. These pairs of PIs adapted, and naive cells exhibit similar sensitivities to treatment, in contrast to those of the BTZ. Furthermore, this compound can overcome adaptation mechanisms on the basis of a reduced load on the proteasome, another clinically relevant form of treatment resistance [3, 4, 232]. These results suggest that the VCP/p97 complex and its cofactors may become a viable therapeutic option for patients with relapsed or PIs-resistant MM.

## Future perspectives

**Fig. 2:**
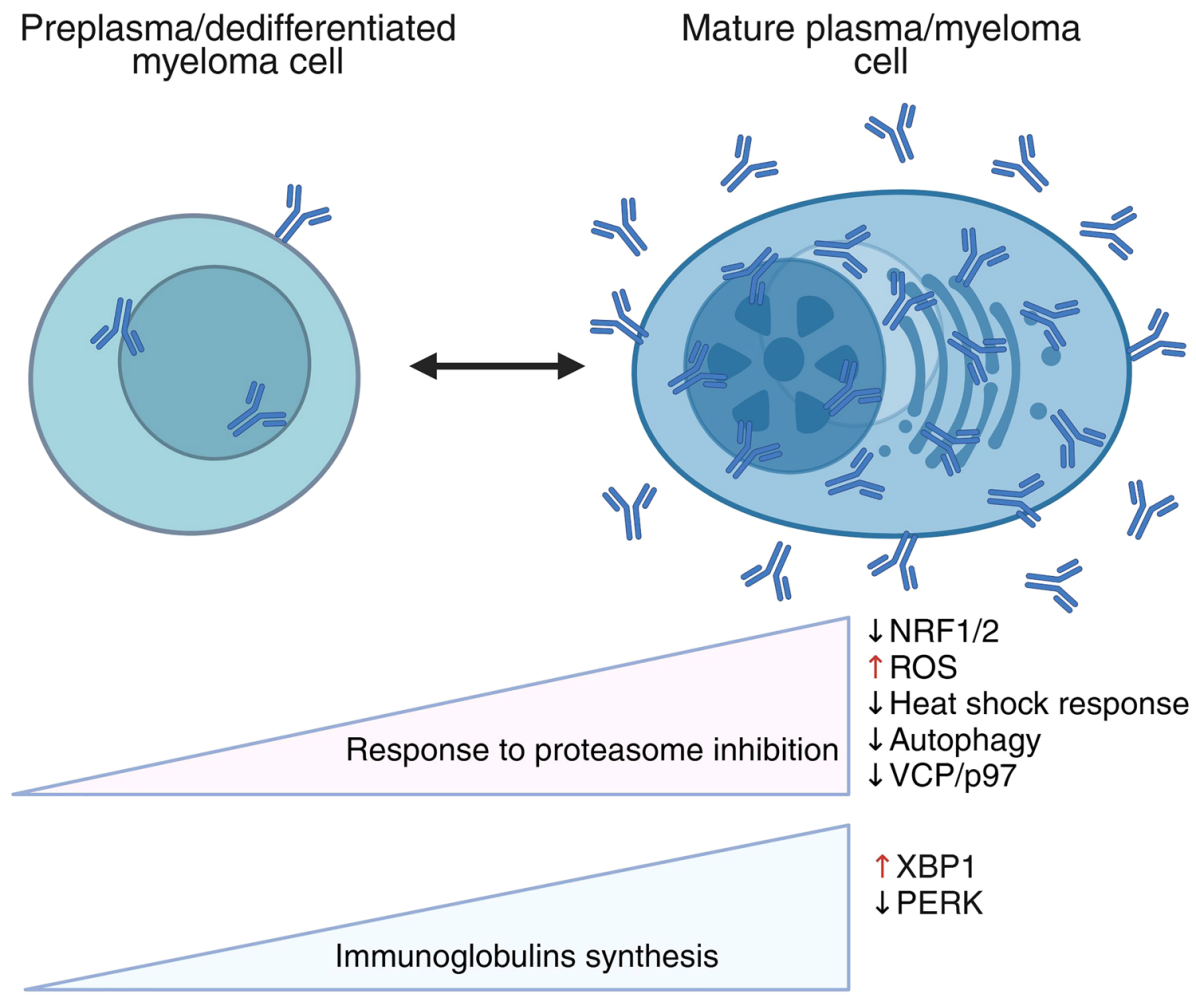
Scheme of the targeting of different parts of proteostasis leading to overloading of protein degradation, which has been evaluated in various types of studies. Created in BioRender (2025)

Herein, I outline considerations focused on the overloading of proteasomes to improve the antimyeloma efficiency of PIs and examine novel therapeutic approaches involving the targeting of other cellular proteostasis participants. Multiple myeloma cells, as well as mature plasma (CD138-positive) cells, display high sensitivity to PIs. In contrast, plasma cells are ephemeral, with limited intense antibody responses, and after a few days of high Ig production, they undergo cell death. High levels of Ig production and genomic alterations make MM cells much more dependent on mechanisms of proteostasis, including protein folding and degradation. Therefore, sensitivity to PIs of cultured or patient-derived cells is correlated with the load to proteasomes (Fig. 2), which explains the high efficiency of PIs against MM and/or mature plasma cells [24, 25, 39]. Protein misfolding can damage cells not only through a loss of function of the original protein but also because of the toxicity caused by protein aggregation. The idea that controlling protein misfolding/proteotoxic stress is a defining feature of cancer cell evolution is interesting. High mutational load cancers, which are comparable to the yeast model, can preserve viability by reducing these harmful effects via proteostasis mechanisms [121, 221, 233].

Proteotoxic stress, as an intrinsic vulnerability of cancer cells, can be enhanced via simultaneous manipulation with more proteostasis mechanisms and offers an attractive solution to attack resistant, relapsed, or refractory MM by taking advantage of their heightened dependence on proteostasis, specifically protein quality control mechanisms. This leads to overload of the protein degradation machinery and to proteotoxicity and subsequent cell death. This dependency is predicted to arise from the level of Ig chain synthesis and the high number of mutations and genomic instabilities [3, 33, 34, 233]. Finally, I consider possible explanations for the apparently limited applicability of PIs and discuss whether simultaneous pharmacological targeting of other components involved in or affecting cellular proteostasis, such as the DDI2-NGLY1-NRF1 axis, redox homeostasis, autophagy, heat shock response, UPR, and p97/VCP, might increase the load to the proteasome and improve PIs action. Therefore, I believe that the proposed approaches offer a direct path to more effective therapy for multiple myeloma or mantle cell lymphoma and provide opportunities for drug repurposing.

## Conclusion

Patients with multiple myeloma do not receive universal treatment, but therapies, such as PIs and immunomodulatory drugs, have improved survival rates globally in the last two decades. Insight into MM pathology is dependent on understanding the protein-handling machinery and plasma cell biology. PIs are effective in multiple myeloma therapies for various reasons, including targeting typical plasma cell features, e.g., a high protein load to the degradation machinery and a strong dependence on the UPR. Therefore, I discuss Ig-producing cell features and interactions between various protein-handling pathways. Unfortunately, drug resistance remains an issue for many patients, particularly for those who relapse or become refractory to these therapies. Further investigation is needed to gain a better understanding of how different parts of proteostasis networks intersect, which reveals new features of resistance and sensitivity against PIs, including other pathways and proteins involved in these processes. The key questions are how to best combine different drugs targeting NRF1, redox homeostasis, HSF1/HSPs, autophagy, PERK, and VCP/p97 with proteasome inhibitors (Table 1) to improve clinical outcomes.

## Data Availability

No datasets were generated or analyzed during the current study.
